# Knowledge, attitudes and perceptions of nursing students regarding vaccines

**DOI:** 10.4102/safp.v66i1.5825

**Published:** 2024-01-31

**Authors:** Mohamed H. Suleman, Saien Govender, Euphemia M. Mhlongo, Keshena Naidoo

**Affiliations:** 1 Nelson R. Mandela School of Medicine, College of Health Sciences, University of KwaZulu-Natal, Durban, South Africa; 2 Centre for the AIDS Programme of Research in South Africa, Durban, South Africa; 3 Department of Nursing, University of KwaZulu-Natal, Durban, South Africa; 4 Department of Family Medicine, University of KwaZulu-Natal, Durban, South Africa

**Keywords:** attitudes, immunisation, knowledge, nursing education, perceptions, vaccines

## Abstract

**Background:**

Final-year nursing students are actively involved in the delivery of public immunisation programmes as part of workplace-based learning, and require adequate knowledge, clinical skills, and attitudes regarding vaccines. This study investigated the knowledge, attitudes and perceptions regarding vaccines of final year nursing students at a South African University.

**Methods:**

This cross-sectional study, through the use of an online survey questionnaire, assessed the knowledge, attitudes and perceptions regarding vaccines and the Expanded Programme of Immunization of final-year nursing students registered at a South African University during the 2021–2022 academic year.

**Results:**

There were 68 participants enrolled in the study (85% response rate). Participants displayed good knowledge regarding vaccines (average score of 52.54/70 ± 5.01 standard deviation [s.d.]), and overall positive perceptions of their training on vaccines and its safety. Knowledge gaps were identified in the mechanisms through which vaccines confer immunity in the human body and the cold chain requirements for the storage of vaccines. Of concern was the prevalent misconception among 78% of participants that vaccines are not effective.

**Conclusion:**

The findings of this study indicate that final year nursing students at the University of KwaZulu-Natal, South Africa have good knowledge regarding vaccines. However, an improved understanding of the mechanism of vaccines will aid nursing students to confront and address misperceptions by clients thereby reducing improving vaccine uptake. Curriculum planners should also consider the inclusion of communication strategies to address vaccine hesitancy.

**Contribution:**

The study contributes to data on nurse education regarding vaccines in the African context, and identifies areas to improve vaccine uptake.

## Introduction

The Expanded Programme of Immunization (EPI) together with other vaccination campaigns recommended by the World Health Organization (WHO) provides a schedule of vaccinations aimed at reducing serious illnesses.^1,2^ The EPI was established in 1974 by the WHO with the objective of ensuring that safe and effective vaccines became available against six major infectious diseases (diphtheria, measles, pertussis, polio, tetanus, and tuberculosis) to all children globally.^3^ The South African Expanded Programme of Immunization (EPI-SA) was officially launched in 1995, and subsequently included hepatitis B vaccine (1995), Haemophilus influenzae type b (Hib) vaccine (1999), pneumococcal conjugate vaccine (2009), and rotavirus vaccine (2009).^4,5^ Immunisation has been one of the most cost-effective public health interventions in South Africa to reduce infant and childhood mortality, as well as to address serious illnesses such as in the recent coronavirus disease 2019 (COVID-19) pandemic. In most developing countries such as South Africa, nurses play a crucial role in administering and providing information about vaccines.^6^ However, vaccine coverage is reported to be sub-optimal.^7^

Public immunisation programmes aim to vaccinate the majority of the population against specific diseases in order to attain herd immunity.^8^ By maintaining vaccination coverage above the threshold for particular diseases, the entire population is protected from those diseases.^9^ Herd immunity thereby protects those who are at risk of infection or are not vaccinated because of their age or health. A national survey revealed that approximately 83.9% of children under 12 months in South Africa receive all their vaccinations. However, despite free vaccination available for children under 5-years-old in South Africa in the public health sector, levels of vaccinations declined to 76.8% among children at age 18 months.^9^ Sub-optimal vaccine coverage has been associated with frequent outbreaks of measles and polio.^10^ A key factor in poor vaccine coverage is vaccine hesitancy.

Vaccine hesitancy refers to a delay in acceptance or refusal of vaccines despite the availability of vaccination services.^11^ Since the onset of the COVID-19 pandemic, there has been widespread misinformation regarding vaccines, thereby increasing vaccine hesitancy among the general population. Increasing vaccine hesitancy among the general population leads to poor vaccination coverage.^12^ Health professionals such as nurses play an important role in providing education and advocacy to the public regarding immunisations, thereby ensuring optimal vaccine uptake and a reduction in vaccine hesitancy.^13,14^ Yet, studies in South Africa report that poor vaccine uptake is associated with a lack of important health information provided by nurses to the public.^15,16^ Nurses’ knowledge and perceptions of vaccines, particularly around vaccine safety, influences the uptake of parenteral vaccines and equally reduces the hesitancy that the general public may experience with regard to vaccine acceptance.^17^ Vaccine knowledge gaps identified among primary care nurses, in the State of Qatar, included the safe handling and administration of vaccines, the efficiency of vaccines, and their contraindications.^18^ A study from Kenya determined that less than a third of nurses had good knowledge and practices regarding adverse events following immunisation, and more than two-third of participants were not knowledgeable about adverse events post-immunisation.^19^

Although immunisation is part of the scope of nurse training, a recent scoping review identified limited inclusion and a lack of training resources for nurse training on immunisation in low- and middle-income countries (LMICs).^20^ It is therefore incumbent on nurse training institutions and educators to evaluate current training, and ensure that nursing students acquire the appropriate knowledge, attitude and skills to deliver effective immunisation services to the community. This study sought to evaluate the knowledge, attitudes and explore perceptions of final year nursing students regarding vaccines. The findings of the study are intended to assist curriculum planners in ensuring that nurse graduates are fit-for-purpose.

## Methods

### Study design

A quantitative cross-sectional descriptive study was conducted using an online survey questionnaire. A quantitative approach is suitable to minimise subjectivity and provide quantifiable results, which can be subject to statistical analysis, graphical representation, and interpretation.

### Setting

The study was conducted among the final-year nursing student population registered at the University of KwaZulu-Natal, South Africa. The 4-year nursing programme includes lectures on vaccines in the third year of the degree programme and practical training at primary care level and at childhood immunisation sites. The study aimed to evaluate the preparedness of pre-licensure nursing students to deliver public health vaccination programmes, thus the study population selected was final year students.

### Sampling

Purposive sampling was applied to recruit participants registered for the 4th year of the nursing degree programme at the University of KwaZulu-Natal between October 2021 and February 2022. Eligible participants were informed of the study by the researchers in person at the university campus. The inclusion criteria entailed participants willing and able to give informed consent, in final year Bachelor of Nursing, over the age of 18 years, registered with the South African Nursing Council (SANC). In all, 68 participants who were enrolled into the study and were provided with the link to the Google Form, where electronic informed consent was obtained and the survey questions were completed.

### Data collection instrument

A previously validated research instrument that was modified for the local context was piloted and refined before data collection ensued. The questions included in the questionnaire were partly based on those in the Dybsand et al. questionnaire; however, additional aspects such as knowledge of the vaccine schedule and administration were original ideas.^21^

The data collection instrument, in English language, captured the sociodemographic characteristics, a quiz to assess knowledge on vaccines, and a five-point Likert scale to elicit attitudes and perceptions around vaccines. The 5 points (‘strongly agree’, ‘somewhat agree’, ‘neither agree nor disagree’, ‘somewhat disagree’ and ‘strongly disagree’), categorise the level of agreement of the participant with the statement on the questionnaire. The knowledge quiz consisted of 70 questions (true-or-false as well as multiple choice questions) and evaluated knowledge on vaccines and vaccine administration, the EPI schedule, and adverse events of vaccinations.^8^

Each correct answer was allocated 1 point for each of the questions in Section B. A combined total score achievable for the knowledge quiz was 70 points. The total scores for each of the sub-sections were as follows: ‘Knowledge of vaccines and immunity’ was 8 points, ‘Knowledge of EPI schedule’ was 37 points, ‘Knowledge of adverse events related to vaccinations’ was 8 points, and ‘Knowledge of administration of vaccines’ was 17 points.

The Likert scale evaluated the participants’ attitudes towards vaccines, perceptions of vaccine safety and efficacy, vaccine hesitancy, and perceptions regarding their training on vaccines. In interpretation of the Likert scale, a quantitative method was used (percentage of students who position themselves in each category). The questionnaire took approximately 10 min to complete.

### Data analysis

Responses to the survey were captured electronically via Google Forms and these responses were transferred to an Excel spreadsheet and imported into Statistical Package for the Social Sciences (SPSS) version 28 (SPSS Inc. Chicago, Illinois, United States), a statistical software programme, for descriptive analysis. Biostatisticians were consulted in the analysis of the data. The data were summarised and analysed using descriptive statistics and frequencies.

### Ethical considerations

Ethics approval was granted by the Humanities and Social Sciences Research Ethics Committee (HSSREC) under reference HSSREC/00003211/2021 at the University of KwaZulu-Natal. Informed consent was obtained from all participants.

## Results

The total number of respondents to the survey was 68 out of a potential 80. This translated to a response rate of 85%.

### Socio-demographic characteristics of participants

The majority of participants (95.6%) fell within the age group of 18 to 24 years old, while only 4.4% fell within the age group of 25 to 34 years old. The majority of participants (79.4%) were female. The majority of the participants (92.6%) were also from African ethnic background, while Indian and mixed race ethnic backgrounds each had a 2.9% participation, and white ethnicity translated to a participation of 1.5%. Only 1.47% of participants were categorised as ‘mature students’, meaning that this group of final-year nursing students had a previous qualification, of which one participant had a social sciences degree (not health-related).

### Student knowledge of vaccines

The knowledge quiz score was out of a total of 70, which was then converted to a percentage. The overall knowledge score of the participants was good with a mean score of 52.54/70 (75%) with a standard deviation (s.d.) of 5.70. The lowest and highest scores were 37 and 67, respectively. Participants possessed significant knowledge regarding administration of vaccines (13.78/17 [81%]), followed by knowledge of the EPI schedule (28.10/37 [76%]), and adverse events following vaccination (5.91/8 [74%]). However, participants displayed acceptable general knowledge of vaccines (4.75/8 [59%]). The results for the vaccine quiz and each of the four components of the vaccine quiz are displayed in Table 1.

**TABLE 1:** Results of four components of knowledge of vaccines quiz.

Section		Mean score	±s.d.	Maximum score	Mean percentage
1.	Knowledge of vaccines and immunity (8 questions)	4.75	±1.297	8	59
2.	Knowledge of EPI schedule (37 questions)	28.10	±4.334	37	76
3.	Knowledge of adverse events related to vaccinations (8 questions)	5.91	±1.181	8	74
4.	Administration of vaccines (17 questions)	13.78	±1.348	17	81

**Total score**		**52.54**	**±5.703**	**70**	**75**

EPI, Expanded Programme of Immunization; s.d., standard deviation.

### Attitudes towards vaccination

**FIGURE 1:**
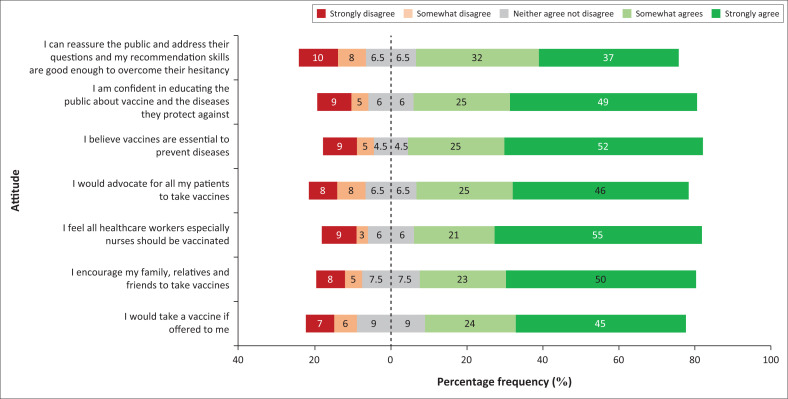
Diverging stacked bar chart showing the percentage frequency of participant attitudes towards vaccines.

Participants indicated that they can reassure the public and address the public’s questions and the nurses’ recommendation skills are good enough to overcome the public’s hesitancy (69%); confident in educating the public about vaccines and diseases they protect against (74%); believe vaccines are essential to prevent diseases (77%); felt all healthcare workers especially nurses should be vaccinated (76%); would advocate for all patients to take vaccines (71%); encourage their family, relatives, and friends to take vaccines (73%); indicated that they would personally accept a vaccine if offered to them (69%). Participants’ responses to seven statements eliciting their attitudes towards vaccinations are summarised in Figure 1.

### Perceptions of safety and efficacy

Nearly 30% of the participants perceive that vaccines are agents that should be administered to patients with a weakened immune system. A third of participants perceive that they are uncertain about whether patients with weakened immune systems should receive vaccines.

Most participants indicated that they perceive the benefits of vaccines outweigh the risks, while less than a third of participants indicated that they are uncertain if they perceive that the benefits of vaccines outweigh the risks.

Regarding the perceptions in relation to the lack of evidence available for the long-term safety of vaccines, less than 50% indicated that they are uncertain if they perceive this to be true or not, while an almost equivalent number of participants indicated that they agree and disagree, respectively, in terms of their perception of a lack of evidence of the long-term safety of vaccines.

Almost half (41%) of participants perceive vaccines as agents to reduce the chances of acquiring life-threatening diseases. More than half of all participants do not perceive vaccines as agents that have serious side effects, while nearly a quarter of participants are not sure if they perceive vaccines to have serious side effects.

**FIGURE 2:**
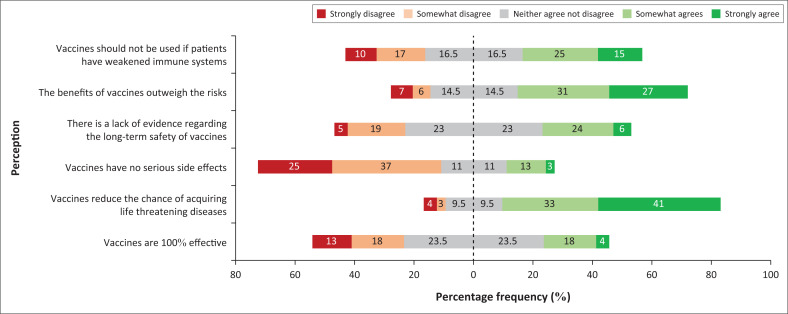
Diverging stacked bar chart showing the percentage frequency of participant perceptions regarding vaccine safety and efficacy.

Less than half of all participants (47%) indicated that they neither agree nor disagree if they perceive vaccines to be 100% effective. A relatively higher number of participants (31%) indicated that they do not perceive vaccines to be 100% effective as opposed to those (22%) who perceive them to be effective (Figure 2).

### Perceptions of vaccine hesitancy

More than 50% of participants perceive that people may be hesitant about vaccines because their fears or concerns are not being addressed, while a third of participants are uncertain whether or not people may be hesitant about vaccines because of their fears or concerns not being addressed.

More than 50% of participants perceive that patients may also be hesitant to accept a vaccine because of insufficient evidence available on the efficacy of vaccines, while more than a quarter of participants perceive that they are not certain if this is in fact the case.

More than 50% of participants perceive that patients do not trust pharmaceutical companies and manufacturers, hence, patients may be hesitant to accept a vaccine, while less than a third of participants are uncertain if they perceive this to be the case.

**FIGURE 3:**
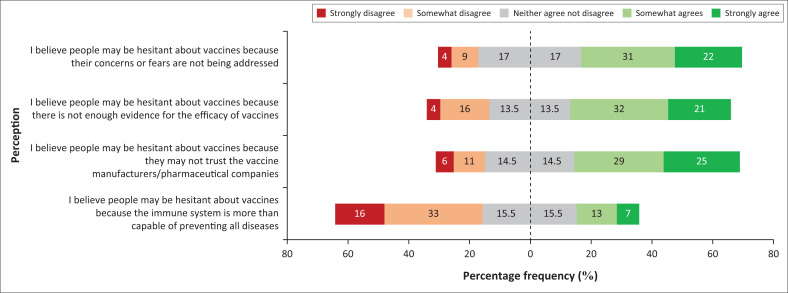
Diverging stacked bar chart showing the percentage frequency of participant perceptions regarding vaccine hesitancy.

Nearly 50% of participants perceive that the immune system is not capable of preventing all types of diseases, while less than a third of participants are uncertain if they perceive the immune system to be capable enough of preventing all types of diseases (Figure 3).

### Perceptions of vaccine education and training

**FIGURE 4:**
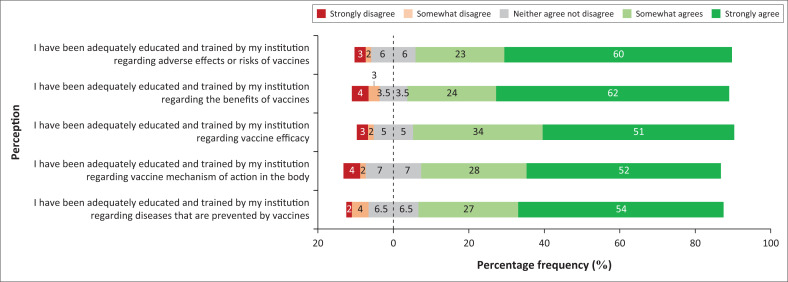
Diverging stacked bar chart showing the percentage frequency of participant perceptions regarding vaccine education.

An overwhelming majority of participants (more than 80%) perceived that the University of KwaZulu-Natal (UKZN) has adequately educated and trained them on (1) which diseases are prevented by vaccines; (2) the mechanism of action of vaccines in the body; (3) vaccine efficacy; (4) benefits of vaccines; and (5) adverse effects or risks of vaccines (Figure 4).

## Discussion

The findings of the study affirmed that final year nursing students possess good knowledge regarding vaccines. Participants were most knowledgeable about administration of vaccines and least knowledgeable about the mechanisms of vaccines and immunity. The findings of this study reflect the emphasis in the nursing curriculum on practical aspects of the vaccine programme and less on basic sciences. Teaching and learning in the nursing programme focus mostly on practical aspect of the immunisation programme. Professional nurses are often responsible for the transportation and administration of vaccines, especially in rural areas and mobile clinics, and a clear understanding of the cold chain requirements is essential to ensure that the vaccines retain their efficacy. However, there was sub-optimal knowledge of the mechanism whereby vaccines confer immunity indicating poor teaching and learning in this area. The explosion of false information about vaccines during the COVID-19 pandemic may also have affected student understanding of immune responses to vaccines. A sound understanding of how immunity is acquired through vaccination may assist nurses in confronting false information in the community and providing the correct information to patients. Some of the strategies identified to improve nursing professionals’ knowledge of vaccines include supportive supervision, educational meetings, and printed educational material.^22,23^

Participants demonstrated strongly positive attitudes towards vaccines. This may be the result of a good foundation of knowledge about vaccines or positive experiences with vaccinations. A study undertaken by Pelly et al. in Canada^24^ showed that a positive correlation exists between vaccine knowledge and having positive attitudes towards vaccines by health profession students, including final year nursing students in Canada.^24^ Participants indicated that they are confident to outline the safety and efficacy of vaccines to the general population. This is important as nurses are on the frontline and often are the first point of contact when vaccination campaigns are underway. The study by Dybsand et al.,^21^ also found high levels of confidence among the undergraduate nursing students with regard to vaccine efficacy, risks of vaccines, vaccine safety, benefits of vaccines, and vaccine immune system burden.^21^ The study conducted by Wilpstra et al. in Canada also found positive attitudes among their undergraduate nursing students in relation to vaccine acceptance.^25^ Another study undertaken Javier et al. in Spain also found positive attitudes among health sciences students and their beliefs with respect to vaccine acceptance.^26^

Pertaining to the safety and efficacy of vaccines, most participants indicated that they perceive that the benefits of vaccines far outweigh any associated risks that accompany it. In contrast, Dybsand et al. identified negative perceptions and opinions among final year nursing students in America regarding vaccine risk to benefit ratio.^21^ This might be because of the lower incidence of infectious diseases in high-income countries (HICs). Outbreaks of preventable diseases such as measles and diphtheria in high HIV-prevalence settings in South Africa are associated with poorer outcomes than in HICs.

Majority of participants also indicated that they believe that vaccine hesitancy may be as a result of people thinking that the immune system is more than capable of preventing all types of diseases. It is therefore likely that participants in this study would advocate for vaccine uptake and actively address vaccine hesitancy. Further research is required to explore the factors for student perceptions and investigate the influence of the hidden curriculum on student perceptions and attitudes regarding vaccinations.

Nearly all participants strongly perceive that UKZN has a strong foundation of education and training with regard to vaccines, in the nursing curriculum (Bachelor of Nursing degree). These findings are similar to the findings of a study conducted by Dybsand et al. in the United States,^21^ wherein it was found that nearly 60% of participants believed that their nursing education and training was adequate with respect to vaccine information.^21^ However, the study conducted by Pelly et al. in Canada^24^ found the opposite to be true. This study concluded that health science students in Canada do not perceive their higher education institution to effectively train students about vaccines and immunisation.^24^ Studies globally report that vaccine advocacy and uptake by nurses was positively influenced by knowledge about vaccines.^27,28^ The promotion of national vaccination programmes is thus affected by nurses’ vaccine knowledge.

*A summary of major findings and shortcomings*: This study found that final year nursing students overall demonstrated good knowledge on vaccines and generally a healthy attitude towards vaccinations. There were divergent views regarding the safety of vaccines. The views around hesitancy were challenging to assess and it appears that there are differing views around reasons for hesitancy. Most participants felt that they had received adequate training around hesitancy.

## Limitations

The study was conducted at a single facility and the findings cannot be extrapolated to other settings. Participant’s perceptions of their teaching and learning on vaccines may not reflect the planned curriculum.

## Conclusion

The findings of this study indicate that final year nursing students at UKZN have good knowledge regarding vaccinations in general. However, there is a need for greater teaching on basic sciences and immunology to empower nurses with the understanding to dispel misperceptions around how vaccines work. An improved understanding of the mechanism of immunity conferred by vaccines will aid nursing students to confront and address misperceptions by clients thereby improving vaccine uptake. Curriculum planners should also consider the inclusion of communication strategies to address vaccine hesitancy.
